# Supplementary material for: Multimodal detection of hateful memes by applying a vision-language pre-training model
Source: PLoS One. 2022 Sep 12;17(9):e0274300. doi: 10.1371/journal.pone.0274300 (PMC9467312; doi:10.1371/journal.pone.0274300)
Supplement: S1 File — (DOCX) [file pone.0274300.s001.docx]

**S1 file. Definitions of hateful message (or hate speech)**

Defining hateful hate messages (or hate speech) is the foundation of our work, yet there are a few candidates competing for this task; to even further obscure our challenge, there is a neighboring category of expression, namely offensive language, that is very similar to hateful messages but being considered more acceptable online. For example, “All Chinese should get out of this place!” is a hateful message while “Get out of this place!” is simply an angry announcement. Therefore, we may first review the definitions from some prominent organizations or individuals to determine the meaning of hateful messages that is distinguishable from that of offensive language:

1. **YouTube, “Hate Speech Policy: YouTube Community Guidelines”**: Hate Speech is not allowed on YouTube. We remove content promoting violence or hatred against members of protected groups including but not limited to ’race, gender, sexual orientation or religious affiliation. We may allow content that includes hate speech like news coverage of world events if their primary purpose is educational, documentary, scientific and artistic in nature.
2. **Facebook, “Community Standard, III. Objectionable Content, Hate Speech”**: We define hate speech as a direct attack against people on the basis of what we call protected characteristics: race, ethnicity, national origin, disability, religious affiliation, caste, sexual orientation, sex, gender identity and serious disease. We define attacks as violent or dehumanizing speech, harmful stereotypes, statements of inferiority, expressions of contempt, disgust or dismissal, cursing and calls for exclusion or segregation. We consider age a protected characteristic when referenced along with another protected characteristic. We also protect refugees, migrants, immigrants and asylum seekers from the most severe attacks, though we do allow commentary and criticism of immigration policies. Similarly, we provide some protections for characteristics such as occupation, when they’re referenced along with a protected characteristic. (<https://www.facebook.com/communitystandards/hatespeech>))
3. **Twitter, “Rules and Policies, Hateful Conduct Policy”**: You may not promote violence against or directly attack or threaten other people based on race, ethnicity, national origin, caste, sexual orientation, gender, gender identity, religious affiliation, age, disability, or serious disease. We also do not allow accounts whose primary purpose is inciting harm towards others based on these categories.
4. **Encyclopedia of American Constitution**: “Hate speech is speech that attacks a person or group based on attributes such as race, religion, ethnic origin, national origin, sex, disability, sexual orientation, or gender identity.
5. **Fortuna et al.**: Hate speech is the language that attacks or diminishes, that incites violence or hate against groups, based on specific characteristics such as physical appearance, religion, descent, national or ethnic origin, sexual orientation, gender identity or other, and it can occur with different linguistic styles, even in subtle forms or when humor is used.
6. **Davidson et al.**: we define hate speech as the language that is used to expresses hatred towards a targeted group or is intended to be derogatory, to humiliate, or to insult the members of the group.

The commonalities of hate speech in these definitions, are their expressed “hatred”, “violence”, “attack”, “threaten”, “diminish”, “humiliation”, “insults” and so on, against “members of protected groups”. And these “protected characteristics” include but not limited to ‘race’, ‘gender’, ‘sexual orientation’ and ‘religious affiliation’. One may refer to section 3.1 for further specifications and examples.

Therefore, we summarize hate speech as such: hate speech is a statement that explicitly or implicitly expresses hatred or violence against people with protected characteristics. This definition distinguishes hate speech from an offensive by their targets: though offensive language can be directed at either individuals or groups, it does not target them due to their protected characteristics. However, this definition alone is not sufficient, as praising can also be a hate speech, i.e., praising KKK or Nazis.

Another challenge for this kind of definition arises when annotating the data: identifying and agreeing whether a specific text is hateful is difficult when annotators are subjective and definitions cannot contain that subjectivity; Ross, et al. studied the reliability of hate speech annotations and suggest that annotators are unreliable. Agreement between annotators, measured using Krippendorff’s *α*, was very low (up to 0.29). However, they also pointed out that the low score is due to their definition not being specific enough. In the following section, we provide further specifications and examples that enable better understandings of hate speech and data labeling.

Twitter, YouTube and Facebook have already provided ample specifications and examples of hate speech on their respective community guidelines web pages; though some of those specifications of hate speech are rather ambiguous and controversial, many of them are clear, illuminating and have already become consensus. Therefore, we integrate those guidelines together to form a more comprehensive and useful instruction that specifies hate speech. Hate speech can be first divided into two general categories: a) hate speech that targets individuals or groups because of their protected characteristics, b) hate speech that doesn’t specify the characteristics of its targets. Next, hate speech can be divided into more subcategories. For the sake of space, the detailed specification is moved to the appendix section. Please see the appendix section for further details.

However, hateful messages should not be confused with profane and offensive messages. Here are the explanations and examples of the aforementioned terms:

1. **Norma**l: When a speech is not considered hate speech, offensive speech, or profane speech, it is normal.
2. **Profanity**: Profanity is a socially offensive use of language, which may also be called cursing, cussing or swearing, cuss words (American English vernacular), curse words, swear words, bad words, dirty words, or expletives. Accordingly, profanity is language use that is sometimes deemed impolite, rude, or culturally offensive. It can show a debasement of someone or something, or be considered an expression of strong feeling towards something. Some words may also be used as intensifiers. Five possible functions of profanity include:
   1. Abusive swearing, intended to offend, intimidate, or otherwise cause emotional or psychological harm; e.g., Go to hell, you damn bast**d!
   2. Cathartic swearing, used in response to pain or misfortune; e.g., I failed my exam, I am f***ed up.
   3. Dysphemistic swearing, used to convey that the speaker thinks negatively of the subject matter and to make the listener do the same; e.g., N***ers are stupid.
   4. Emphatic swearing, intended to draw additional attention to what is considered to be worth paying attention to; e.g., This show is damn f***ing cool!
   5. Idiomatic swearing, used for no other particular purpose, but as a sign that the conversation and relationship between speaker and listener is informal; e.g., These are my ni***.
3. **Offensive**: Offensive language is similar to hate speech; it acts against the target; yet it doesn’t contain strongly malignant intent against the target; e.g., “Get out of this place!” “F*** off!”

**(**Refer to website: [openprofanitylist.com/Download/List](http://openprofanitylist.com/Download/List) for more examples.)

Hate speech promotes violence or hatred against individuals or groups based on any of the following **target classes** of protected characteristics:

1. **Age**
2. **Caste**
3. **Disability**
4. **Ethnicity**
5. **Gender Identity and Expression**
6. **Nationality**
7. **Race**
8. **Immigration Status**
9. **Religion**
10. **Sex/Gender**
11. **Sexual Orientation**
12. **Victims of a major violent event and their kin**
13. **Veteran Status**
14. **No specific target**

Hate speech against the members of these groups contains the following categories of actions:

1. **Violent Threats**

Hate speech that contains violent threats against an identifiable target. Violent threats are declarative statements of intent to inflict injuries that would result in serious and lasting bodily harm, where an individual could die or be significantly injured, e.g., “I will kill you.” Note that threats such as “Get off or I would kick your ass!” would not be considered hate speech, though they are offensive. At the same time, this definition applies whether or not the target is with protected categories.

1. **Calling Serious Harms**

Hate speech contains wishing, hoping or calling for serious harm on a person or group of people. This includes, but is not limited to:

- 1. Hoping that an entire protected category and/or individuals who may be members of that category die as a result of a serious disease, e.g., “I hope all [nationality] get COVID and die.”
  2. Wishing for someone to fall victim to a serious accident, e.g., “I wish that you would get run over by a car next time you run your mouth.”
  3. Saying that a group of individuals deserves serious physical injury, e.g., “If this group of [slur] don’t shut up, they deserve to be shot.”
  4. Encouraging others to commit violence against an individual or a group based on their perceived membership in a protected category, e.g., “I’m in the mood to punch a [racial slur], who’s with me?”

1. **Hateful Slurs as Degradation** Hate speech that targets individuals or groups with repeated slurs, tropes or other content that intends to dehumanize, degrade or reinforce negative or harmful stereotypes about a protected category. This includes repeated and/or non-consensual slurs, epithets, racist and sexist tropes, or other content that degrades someone.
2. **Incitement Against Protected Categories** Hate speech that intends to:
   1. incite fear or spread fearful stereotypes about a protected category, including asserting that members of a protected category are more likely to take part in dangerous or illegal activities, e.g., “all [religious group] are terrorists.”
   2. to incite others to harass members of a protected category on or off the platform, e.g., “I’m sick of these [religious group] thinking they are better than us, if any of you see someone wearing a [religious symbol of the religious group], grab it off them and post pics!”
   3. to incite others to discriminate in the form of denial of support to the economic enterprise of an individual or group because of their perceived membership in a protected category, e.g., “If you go to a [religious group] store, you are supporting those [slur], let’s stop giving our money to these [religious slur].”
3. **Dehumanilzation**

Hate speech that degrades the members of these groups in the form of comparisons, generalizations or unqualified behavioral statements to or about:

- 1. Insects
  2. Animals that are culturally perceived as intellectually or physically inferior
  3. Filth, bacteria, disease and feces
  4. Inferior humans
  5. Sexual predators, thieves, bank robbers, and other criminals Specific examples include:

1. Black people and apes or ape-like creatures
2. Black people and farm equipment
3. Caricatures of Black people in the form of blackface
4. Jewish people and rats
5. Jewish people running the world or controlling major institutions such as media networks, the economy or the government
6. Denying or distorting information about the Holocaust
7. Muslim people and pigs
8. Muslim person and sexual relations with goats or pigs
9. Mexican people and worm-like creatures
10. Women as household objects or referring to women as property or “objects”
11. Transgender or non-binary people referred to as “it”
12. Dalits, scheduled caste or “lower caste” people referred to as menial laborers
13. **Denial of existence**

Hate speech that asserts the non-existence of the individual or groups with the aforementioned classes of protected characteristics, or an event that hurts some groups of people is non-existent

1. **Insulting Victims**

Hate speech that mocks, insults, harass, or deny the existence of, the concept, events or victims of mass murder, violent events, or specific means of violence

1. **Exaggerating Inferiority**

Hate speech that exaggerates physical deficiencies, mental deficiencies and moral deficiencies of the protected groups by degrading them with derogatory words:

- 1. Derogatory terms related to sexual activity, including, but not limited to: whore, slut, perverts
  2. Expressions about being less than adequate, including, but not limited to: worthless, useless
  3. Expressions about being better/worse than another protected characteristic, including, but not limited to: “I believe that males are superior to females.”
  4. Expressions about deviating from the norm, including, but not limited to: freaks, abnormal

1. **Support of Hate Crime** Hate speech includes the following types of actions that target the protected groups:
   1. Supporting groups that commit hate crimes, including but not limited to: supporting Nazi, supporting genocides
   2. Supporting segregation in the form of calls for action, statements of intent, aspirational or conditional statements
   3. Supporting explicit economical, social and political exclusion, by denying access to economic entitlements and limiting participation in the labor market, denying access to physical and online spaces and social services, and denying the right to political participation
   4. Promoting hateful logos, symbols, imageries and sayings of hateful groups
   5. Self-admission to intolerance on the basis of protected characteristics, including, but not limited to: homophobic, islamophobic, racist
   6. Expressions that a protected characteristic shouldn’t exist
   7. Expressions of hate, including, but not limited to: despise, hate
   8. Expressions of dismissal, including, but not limited to: don’t respect, don’t like, don’t care for
   9. Expressions suggesting that the target causes sickness, including, but not limited to: vomit, throw up
   10. Expressions of repulsion or distaste, including, but not limited to: vile, disgusting, yuck
       1. **what are considered offensive languages instead of hate speech**
          1. Referring to the target as genitalia or anus, including, but not limited to: cunt, dick, asshole
          2. Profane terms or phrases with the intent to insult, including, but not limited to: fuck, bitch, motherfucker
          3. Terms or phrases calling for engagement in sexual activity, or contact with the genitalia, anus, feces or urine, including but not limited to: suck my dick, kiss my ass, eat shit
          4. Content that describes or negatively targets people with slurs, where slurs are defined as words that are inherently offensive and used as insulting labels for the above-listed characteristics.
